# Burnei’s anterior transthoracic retropleural approach of the thoracic spine: a new operative technique in the treatment of spinal disorders

**Published:** 2015

**Authors:** TS Gavriliu, EM Japie, RA Ghiță, Ș Hamei, C Dughilă, IL Țiripa, T Elnayef

**Affiliations:** * ”M. S. Curie” Children’s Clinical Emergency Hospital, Bucharest, Romania; ** ”Carol Davila” University of Medicine and Pharmacy, Bucharest, Romania

**Keywords:** anterior approach, thoracotomy, thoracoplasty, retropleural, diskectomy

## Abstract

**Background:** Up to the middle of the last century, the thoracic spine, especially in its upper part, has been considered an unapproachable site, a no-man’s land, but the constant evolution of medicine imposed techniques of the spine at these levels in order to solve a large area of pathology (infectious, tumoral, traumatic, and last but not least, deformative). This way, a series of anterior approaches allowed surgeons to gain access to the anterior part of the spine and the posterior mediastinum. The approaches described by Hodgson, Mirbaha or transthoracic transpleural approach (T4-T11), are enumerated. The idea to allow a more visible and extensive approach, but to avoid respiratory issues due to the lesion of the pleura, led to the description of a new anterior approach by Burnei in 2000.

**Material and method:** Burnei’s approach represents an anterior approach to the thoracic spine, being a transthoracic and retropleural one. This approach allows a large area of spinal pathology due to infectious, traumatic, tumoral and degenerative (idiopathic or congenital scoliosis) causes. Statistically, this approach has been performed more frequently in cases of spinal instrumentation after diskectomy, in order to perform a partial correction of severe, rigid idiopathic scoliosis with more than 70 degrees Cobb and in cases of congenital scoliosis for hemivertebra resection and somatic synthesis to correct the scoliotic curve.

**Results:** This kind of anterior approach allows the surgeon a large visibility of the anterior thoracic spine, diskectomies of up to 5 levels to tender the curve of the deformity and to ensure somatic or/ and transpedicular synthesis of up to 6 thoracic vertebrae. By performing a thoracotomy involving the resection of the posterior arches of the ribs, a thoracoplasty is also ensured with functional and aesthetic effects, by ameliorating the thoracic hump due to the scoliotic deformity.

**Conclusions:** Burnei’s approach joins all the other anterior approaches of the spine, addressing a large area of pathology of the thoracic spine. Even if difficult to be performed, requiring a thorough and perfect technique in the hands of a skilled surgeon, it will ensure satisfaction due to the detailed and visible exposure of the thoracic spine.

## Definition

Burnei’s approach represents an anterior transthoracic retropleural approach addressing to T4-T11 region of the spine. This approach resides in a unilaterally resection of up to 6 ribs (a thoracotomy) in order to expose the majority of vertebral parts (vertebral bodies, intervertebral disks, pedicles and posterior parts of the spine on the side of the approach) and neural parts (medullary canal and nerve roots), but also to allow somatic and/ or transpedicular instrumentation of the spine. 

## Introduction

The anterior approaches of the spine have been used in the medical practice since the middle of the last century. From the historical point of view, the first to describe an anterior approach, transpleural and retroperitoneal (T9-L5), in the treatment of spinal tuberculosis, were Hodgson and Stock, in 1956 [**1**]. In 1969, Dwyer [**2**] described an anterior approach in order to treat scoliosis and Harrington [**3**] stabilized the anterior spine following fracture on tumoral bone with orthopedic cement. In order to get access to the anterior part of the thoracolumbar junction (T11-L5), in 1973, Mirbaha [**4**] described a retroperitoneal extrapleural approach. Next, these approaches became popular and have been used for a large area of pathology of the spine: infections, tumors, trauma and spinal deformities. In the last decades, the anterior approach has made part of the arsenal of the spinal surgeon in the correction of scoliotic deformities in the thoracic and thoracolumbar area. The choice of an anterior approach of the spine to correct the deformity relies on a series of factors concerning the deformity’s nature, localization and surgeons’ preference. This way, the surgeon is able to perform a spinal fusion on a smaller spinal area, ensuring a higher mobility of the spine. Anterior instrumentation is able to ensure a large and strong correction, even if it represents a more difficult approach than the standard posterior one. 

## Indications

Burnei’s approach may be used in spinal pathology such as:

- Idiopathic scoliosis;

- Congenital scoliosis;

- Thoracic Kyphosis;

- Spinal infections;

- Thoracic tuberculosis;

- Some spinal trauma (fracture-dislocation, fracture by compression);

- Some tumors of the thoracic spine (primitive, metastatic or vicinity).

This approach allows the following:

- Convex diskectomy and hemiepiphysiodesis;

- Instrumentation and correction of idiopathic curves;

- Resection of hemivertebrae and somatic instrumentation in congenital scoliosis;

- Posterior instrumentation after diskectomies and hemiepiphysiodesis: 5-6 screws to partially correct severe, rigid scoliosis with more than 70 degrees Cobb;

- Anterior approach of thoracic vertebrae and posterior transpedicular instrumentation of 5 vertebrae (apical and 2 levels below and above the apex). After the somatic approach, the extension of the incision with 2 cm cranially and posteriorly allows the placement of screws on the opposite side.

**Surgical technique**

**Fig. 1 a,b:**
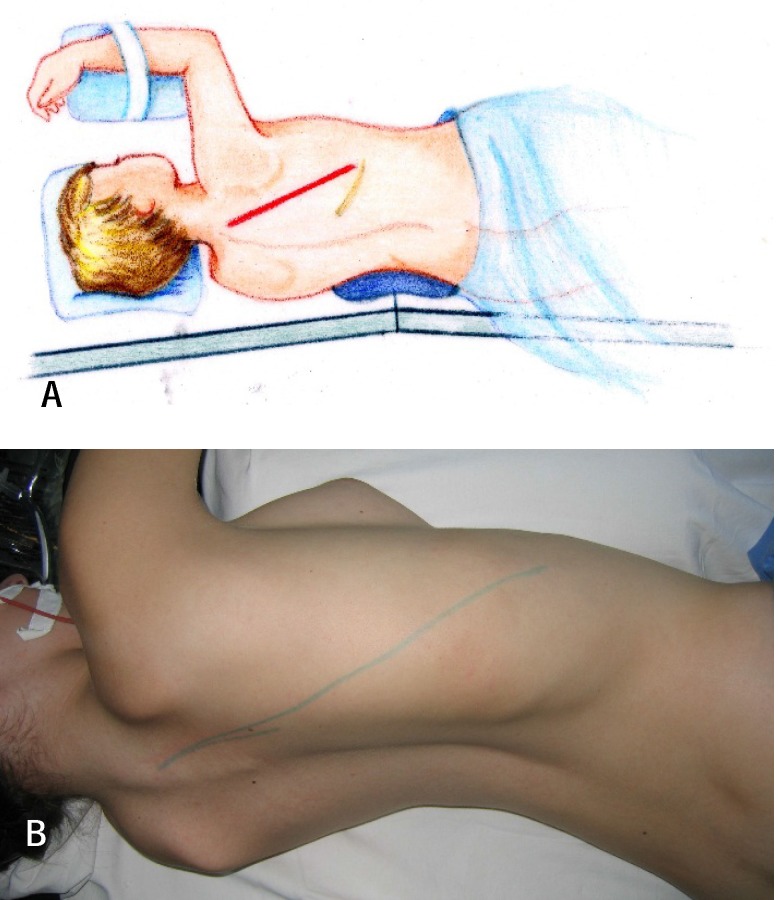
Positioning of the patient in lateral decubitus

**1.Preoperative positioning.** The patient is positioned in lateral decubitus, slightly inclined towards anterior, with the apex of the deformity or area to be addressed upwards (**Fig. 1 a,b**).

**Fig. 2:**
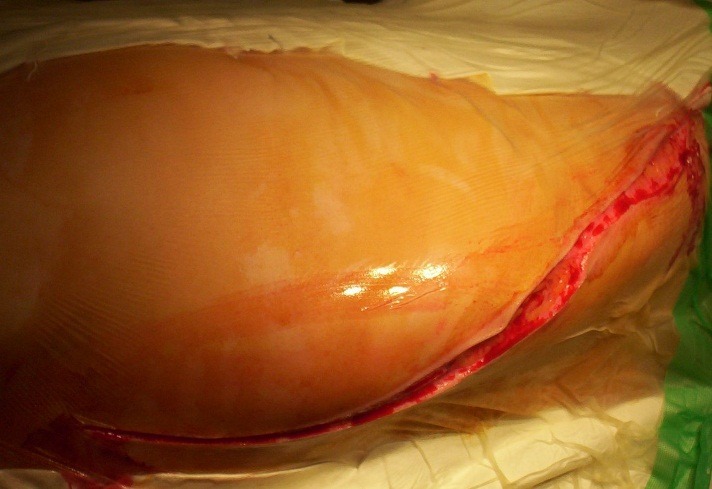
Oblique incision on the posterolateral hemithorax starting 2 cm lateral of the spinous process down to the mid-point of the XIIth rib

**2. The incision**. Oblique incision on the posterolateral wall of the hemithorax, starting from 2 cm laterally of the spinous apophysis to the XIIth rib’s mid-point (**Fig. 2**).

**Fig. 3 a,b:**
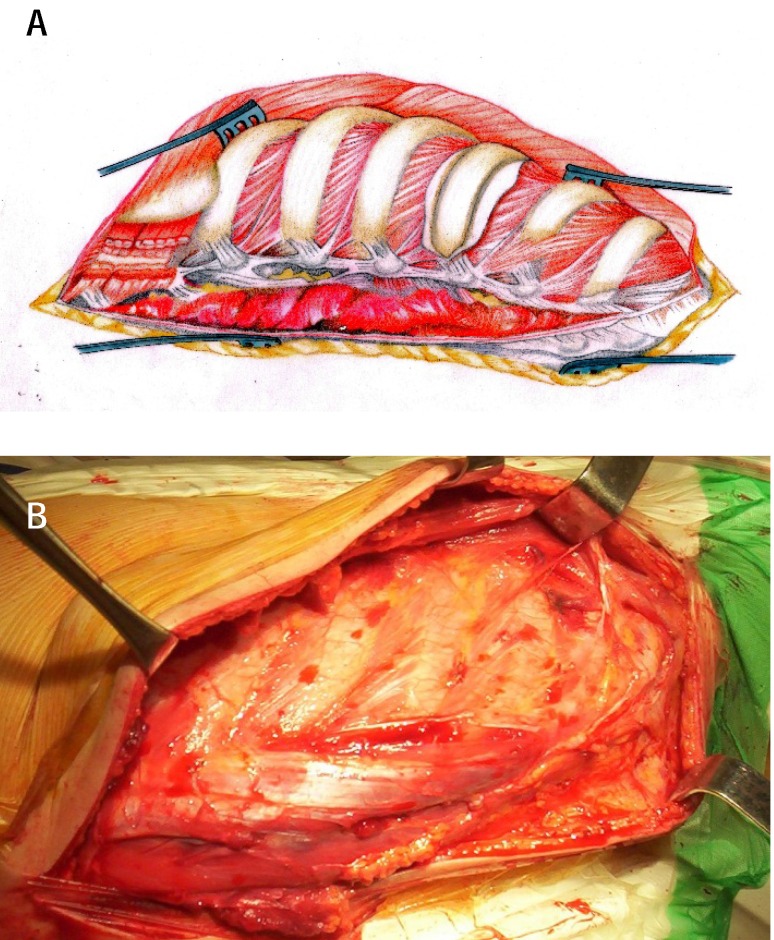
The exposure of the ribs and longitudinal sectioning of the paravertebral muscle layer (iliocostal, longissimus and spinal muscles) in order to protect them and to expose the spine and costotransverse joints

**Fig. 4 a,b:**
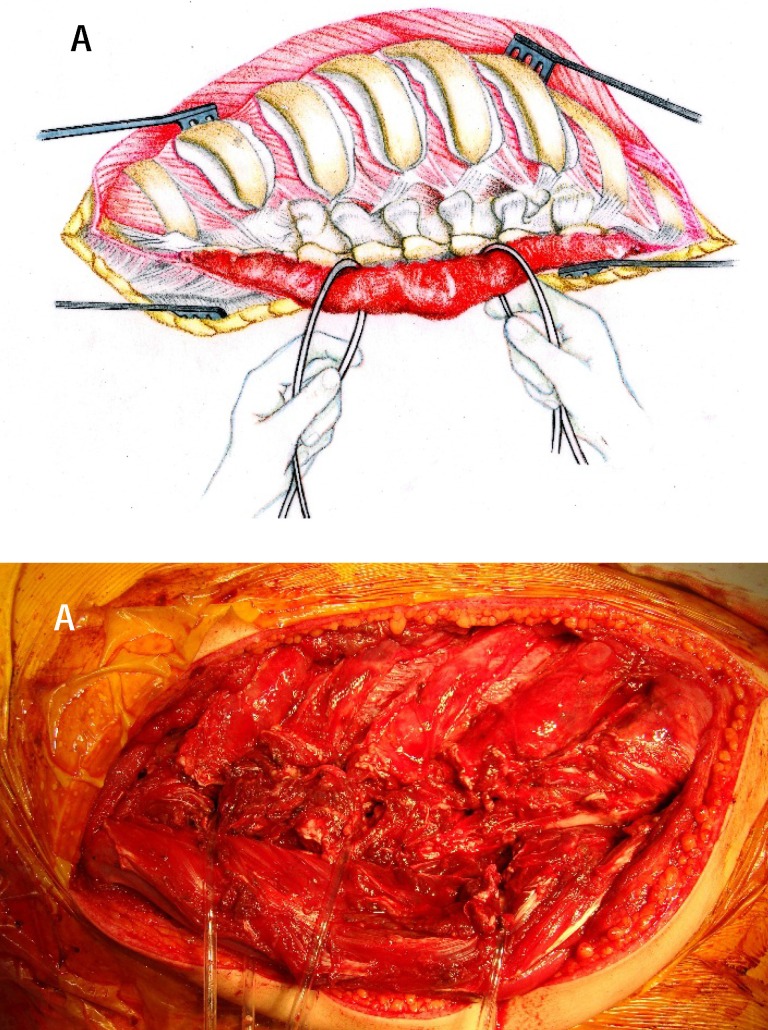
The exposure of transverse processes, of vertebral laminae, of spinous facets on the side of the approach by placing the paravertebral muscles on two loops

**3. Muscular plan dissection** to the ribs and visual identification of the ribs that must be resected. The following muscles are to be dissected: trapezius, latissimus, serratus anterior, partially rhomboideus and iliocostal, longissimus and spinalis muscles that will be protected. The cutaneous-subcutaneous flap is lifted towards the posterior median line to the spinous apophyses. A thorough hemostasis allows an easy recognition of the anatomical fixed points. The lateral ridge of the spinal muscle is clearly shown inside the surgical site. The muscle is dissected 2 cm laterally from the ridge (**Fig. 3 a,b**) and it is lifted with a Cobb elevator, revealing the transverse apophyses, the costotransverse joints, the vertebral laminae, and the same-sided vertebral spinous apophyseal facets. The spinal muscles are isolated with 2 loops placed proximally and distally (**Fig. 4 a,b**).

**4. The thoracoplasty.** The surgeon exposes the most prominent rib, generally found on the convex side, which matches the curve’s apex. The other 2 or 3 proximal and distal ribs are then resected in the same manner. It is important not to resect more than 6 ribs simultaneously in order to preserve the respiratory dynamics. The periosteum is dissected from the rib’s midline to the costotransverse joint and it is then removed from the rib’s anterior aspect in order to identify the 2 muscular “sprouts” and, consequently, the spinal muscles’ insertion areas. The guiding sutures are also attached to the periosteum. The cranial and caudal rims of the rib are carefully resected with an elevator or blunt dissector, paying attention not to damage the pleura. The rib’s cranial rim is dissected from the lateral arch in a posterior direction, while the caudal rim is excised anteriorly. The dissection is completed with a triangular elevator. The rib is cut on the midline, and the posterior part is lifted in order to dissect the costotransverse ligaments. The endothoracic fascia and pleura are detached, exposing the neck and head of the rib. The rib is ablated by rotation and translation moves. The rib that matches the curve’s apex is the easiest to remove.

**Fig. 5 a-c:**
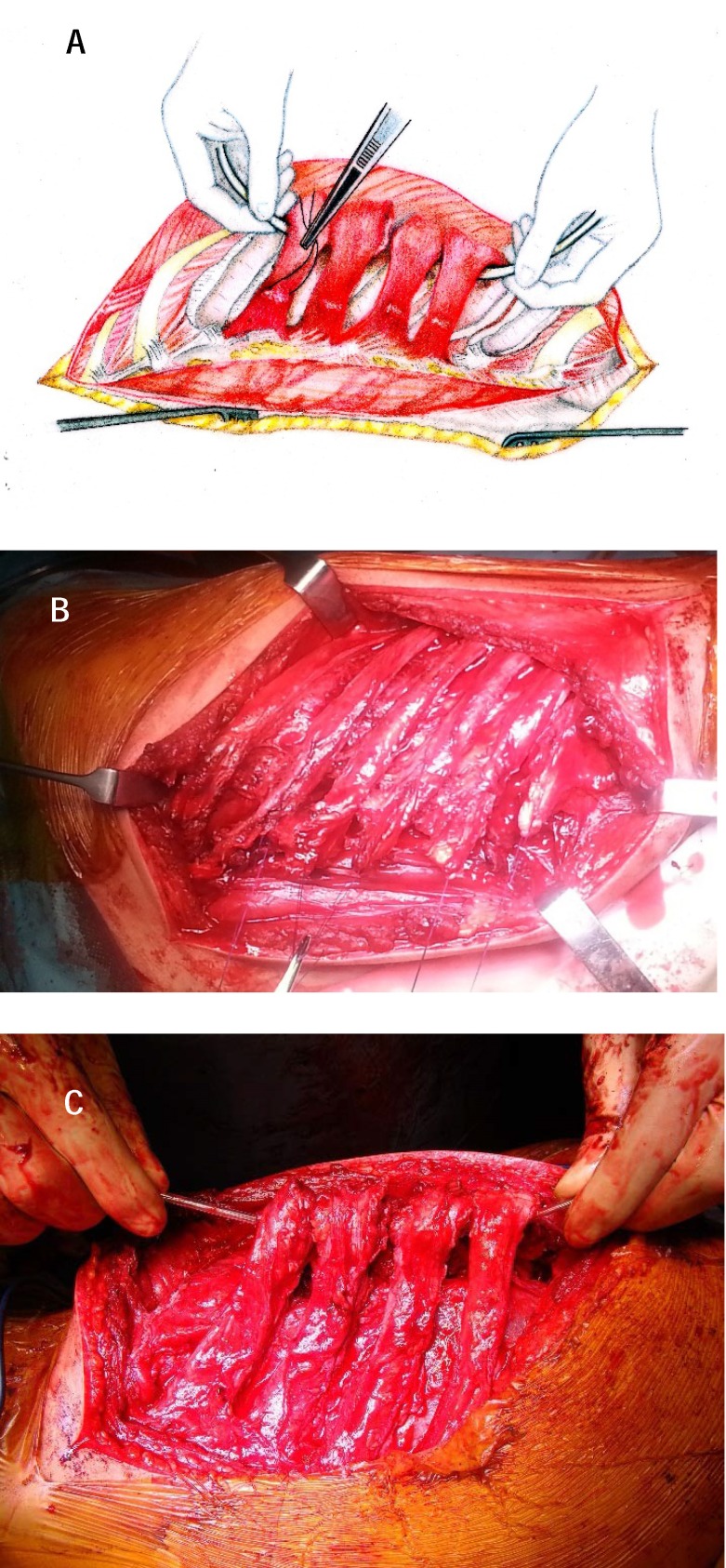
The exposure of the latero-somatic spaces after rib resections, the intercostal nervous and vascular bundles being lifted and protected by a loop. The muscular sprouts are identified and placed on suture anchors to mark the reinsertion area of the spinal muscles

**Fig. 6 a-c:**
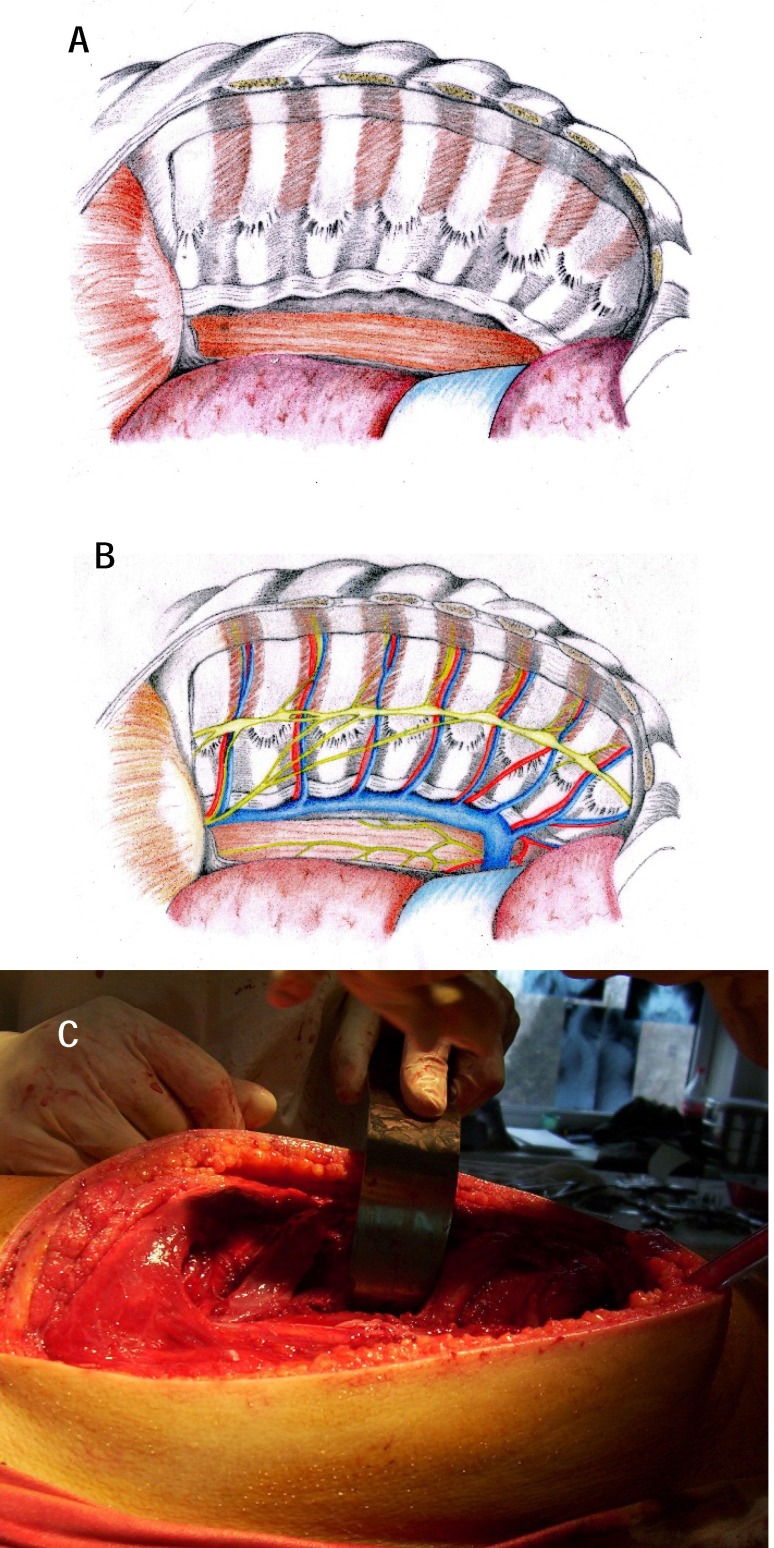
Exposure of the retropleural space and posterior mediastinum which will be protected

The 5 or 6 real latero-somatic spaces can be unified by digital detachment, forming a continuous longitudinal space which may be extended antero-somatically, allowing a digital control in the adjacent latero-somatic space. The muscles and vasculonervous intercostal bundles are lifted and protected by a loop (**Fig. 5 a-c**). The surgeon must be very careful to protect the posterior mediastinal structures by using retractors (**Fig. 6 a-c**), and the transverse segmentar vessels are ligated only if needed.

**Fig. 7 a,b:**
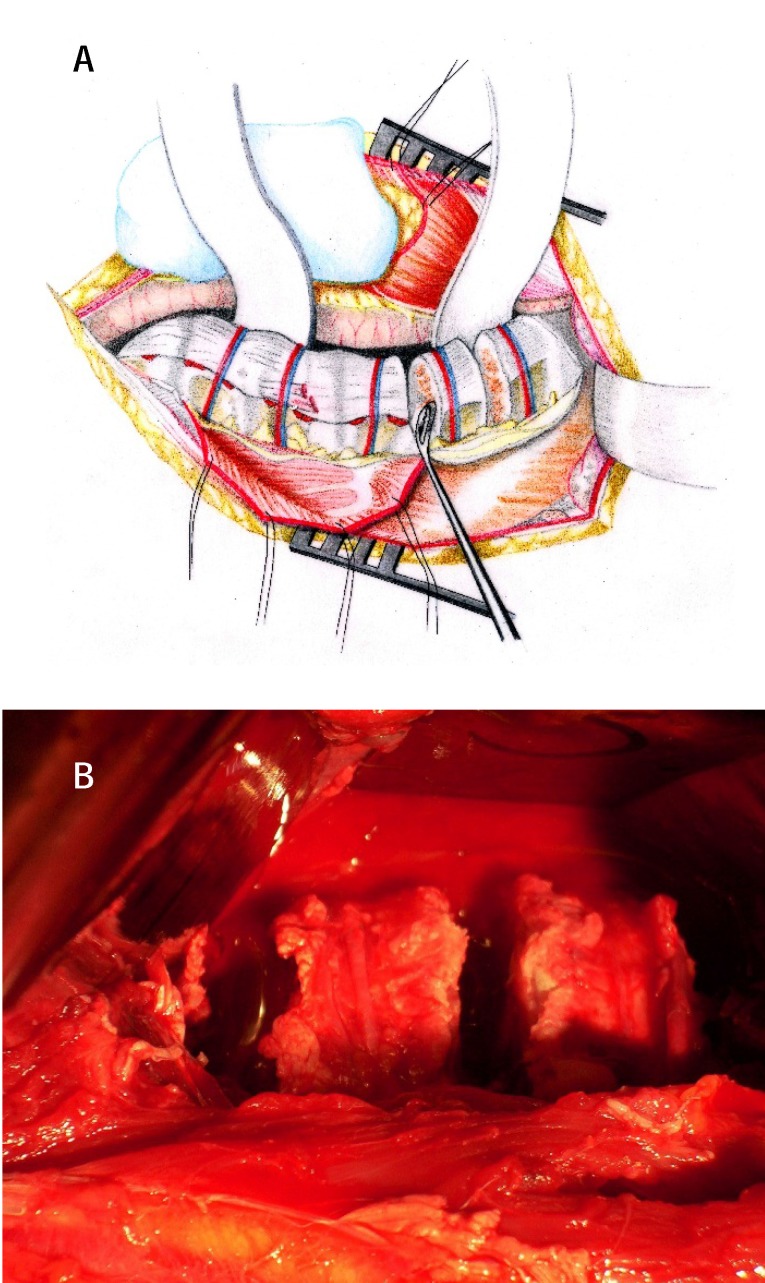
The performing of diskectomy, the ligation of the segmentar vertebral vessels not being a necessity

**5.Diskectomy.** In order to supple the spine and to obtain a maximal correction, diskectomy is performed on up to 5 levels (**Fig. 7 a,b**).

**Fig. 8 a-c:**
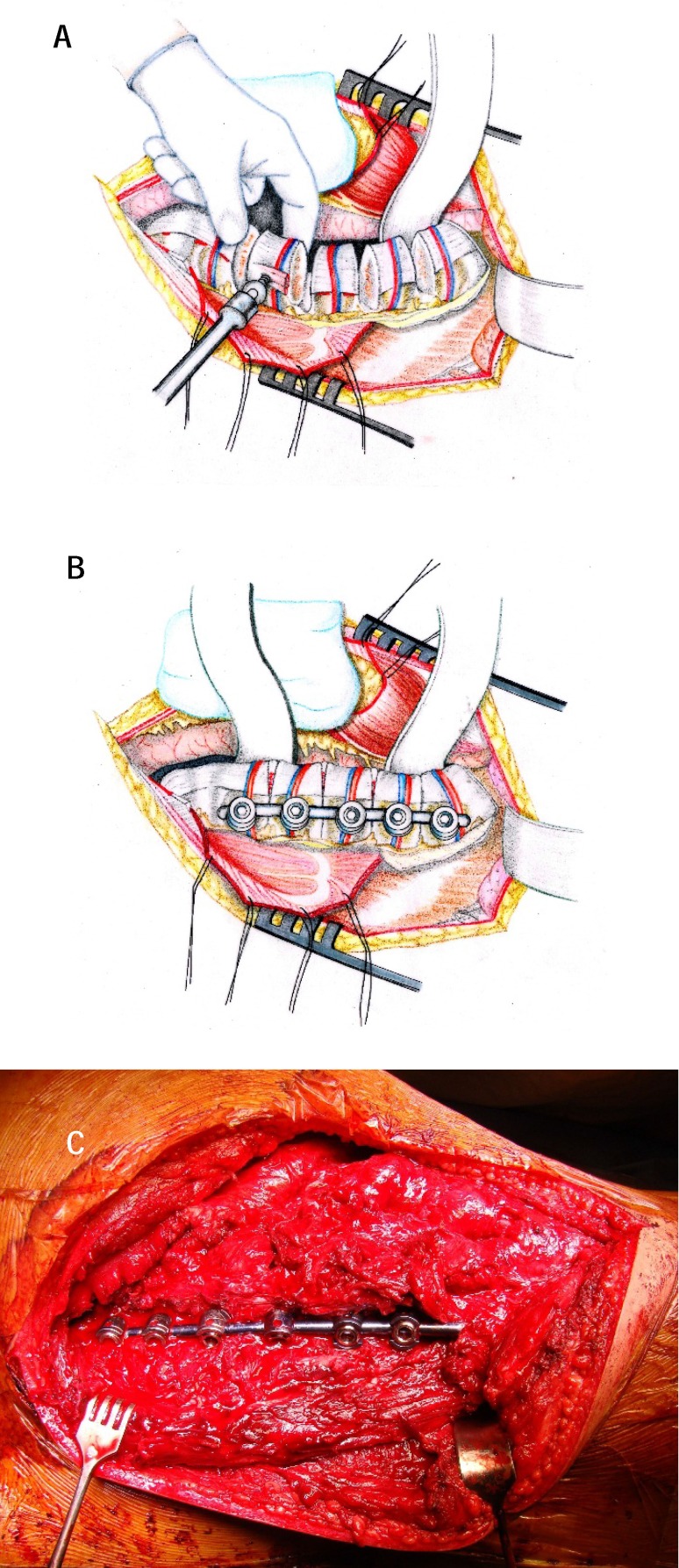
Somatic instrumentation and curve correction on spinal implant

**6.Instrumentation.** The large retropleural exposure of 2-3 upper and 2-3 lower vertebrae related to the apical vertebra allows somatic and/ or transpedicular instrumentation. The same approach was used to resect a vertebra or a spinal segment (**Fig. 8 a-c**).

**7.Observations**. If the periosteum on the pleural face of the rib is dissociated along when introducing the retractors, it is not necessary to repair it.

Pleural lesions require complete repair and drainage of the pleural cavity. The closure of the approach requires rigorous hemostasis and paravertebral drainage for 2-3 days.

External periosteal layers of the ribs are sutured and the spinal muscles are reattached to the marked sites. The muscular thoracic layers are reattached anatomically and the skin is intradermically closed.

## Complications

Potential intraoperative complications are represented by vascular lesions (aorta, cava or Adamkiewicz’s artery), neurological lesions (medulla, thoracic segmentar nerve roots), thoracic duct, pleura or lung lesions.

## Discussions

Spine surgery has undergone a major revolution in the last decades of the last century, technology booming and requiring a large area of access points to the spine. That is why the progress of surgical techniques and spinal instrumentation dramatically reduced morbidity and mortality due to spinal issues and the anterior approaches became first intention in many clinical situations. The main disadvantage in using an anterior approach resides in a potential pathology due to a thoracotomy or thoracoplasty and the need to associate a further surgical procedure to decompress the medulla or to perform a posterior instrumentation. This is why we remarked the use of Burnei’s approach as a real advantage, which allows both somatic and transpedicular instrumentation, even if on a limited area, simplifying the secondary posterior procedure.

The classic posterior approach is impaired by a longer surgical time, with significant blood loss and postoperative pain. In addition, the anterior approach lessens the lesions of the posterior vertebral ligaments and segmental nerve roots, with an earlier mobilization of the patient [**5**].

In contrast to the posterior approach, this anterior approach allows a direct access to the lesion, a great visibility of the anterior parts of the spine, vertebral bodies, intervertebral disks, spinal canal, nerve roots, the only unapproachable structures being the pedicles and posterior vertebral structures on the opposite side. The risks of infection are less in comparison to the posterior approach [**6**]. 

## Conclusions

Burnei’s approach rallies to the other types of anterior approach of the thoracic spine, a diversity of spinal pathology being addressed. Even if it is difficult to be performed, requiring a thorough and perfectly performed surgical technique, in the hands of a skilled surgeon it confers a large and detailed exposure of the thoracic spine.

**Acknowledgements**

This paper was done inside The Sectorial Operational Program for Human Resources Development. POSDRU/159/1.5/S/137390
